# Monitoring Technoscientific Issues in the News

**DOI:** 10.1007/978-3-030-65965-3_37

**Published:** 2020-12-09

**Authors:** Alberto Cammozzo, Emanuele Di Buccio, Federico Neresini

**Affiliations:** 5 University of Sydney, Sydney, NSW Australia; 6 Monash University, Clayton, VIC Australia; 7 University of Bari Aldo Moro, Bari, Italy; 8 University of Bari Aldo Moro, Bari, Italy; 9 University of Guelph, Guelph, ON Canada; 10 University of Caen Normandy, Caen, France; 11 University of Pisa, Pisa, Italy; 12 Norwegian University of Science and Technology, Trondheim, Norway; 13 University of Porto, Porto, Portugal; 14 UPC BarcelonaTech, Barcelona, Spain; 15 University of Porto, Porto, Portugal; 16 Fraunhofer IAIS, St. Augustin, Germany; 17 Royal Holloway University of London, Egham, UK; 18 University of Lisbon, Lisbon, Portugal; 19 University of Bari Aldo Moro, Bari, Italy; 20 University of Lisbon, Lisbon, Portugal; 21 University of Bari Aldo Moro, Bari, Italy; 22 ICAR-CNR, Rende, Italy; 23 University of Naples Federico II, Naples, Italy; 24 University of North Carolina, Charlotte, NC USA; 25 Australian National University, Canberra, ACT Australia; 26 Leibniz University Hannover, Hannover, Germany; 27 Technical University of Dortmund, Dortmund, Germany; 28 University of Southern Denmark, Odense, Denmark; 29 University of Pisa, Pisa, Italy; 30 Warsaw University of Technology, Warsaw, Poland; 31 ISTI-CNR, PISA, Italy; 32 Berlin Institute of Technology, Berlin, Germany; 33 Berlin Institute of Technology, Berlin, Germany; 34 Norwegian University of Science and Technology, Trondheim, Norway; 35 Department of Philosophy, Sociology, Education and Applied Psychology, Section of Sociology University of Padova, Padova, Italy; 36 Department of Information Engineering, University of Padova, Padova, Italy; 37 Department of Statistical Sciences, University of Padova, Padova, Italy

**Keywords:** Media monitoring, News analytics, Computational social science

## Abstract

Research at the intersection between Science and Technology Studies (STS) and Public Communication of Science and Technology (PCST) investigates the role of science in society and how it is publicly perceived. An increasing attention has been paid to coverage of Science and Technology (S&T) issues in newspapers. Because of the availability of a huge amount of digitized news contents, the variety of the issues and their dynamic nature, new opportunities are offered to carry out STS and PCST investigations. The main contribution of this paper is a methodology and a system called TIPS that was co-shaped by sociologists and computer scientists in order to monitor the coverage of S&T issues in the news and to study how they are represented. The methodology relies on machine learning, information retrieval and data analytics approaches which aim at supporting expert users, e.g. sociologists, in the investigation of their research hypotheses.

## Introduction

Science and Technology (S&T) have a promising role in the contemporary society: their contribution is often considered crucial to solve issues that are collectively acknowledged, such as climate change, energy supply or waste management. One problem is that when S&T issues become publicly relevant, the way they are discussed or they are represented can be very different from the “specialist” perspective. The study of these representations could be useful to provide policy makers with insights on the public perception of some issues on which they should or intend to take actions, or provide guidance on the way these issues should be publicly discussed (e.g. on the use of “sensible” words or aspects related to the issues).

The main contribution of this paper is a methodology and a system that were co-shaped by sociologists and computer scientists to support the investigation on this research direction at the intersection between Science and Technology Studies (STS) and Public Communication of Science and Technology (PCST). Previous interdisciplinary studies in PCST have been carried out, either adopting a content analysis perspective, e.g. [21], or following a network analysis, e.g. [10]. Differently from those works, this paper focuses on newspaper articles to investigate the perception of technoscientific issues in the public sphere. “Technoscience” is a useful neologism coniated by STS scholars in order to indicate the increasingly fusion of science and technology [11]. Even if a great deal of attention has been recently paid to social media, newspapers are still very attractive sources because they allow broader longitudinal studies, reach a wide audience, and their content can be regarded reasonably as a proxy of those spread by other media.

This work reports on the design and the implementation of a methodology allowing to monitor and analyse technoscience coverage in online newspapers.

## Background and Methodological Problems

The work reported in this paper has been carried out as part of an interdisciplinary research project called TIPS: Technoscientific Issues in the Public Sphere. The project is interdisciplinary because it involves sociologists, computer scientists, linguists, psychologists and statisticians. The objective of the TIPS project is the analysis of the presence of technoscience in the mass media, which constitute a relevant part of the public sphere. These analyses are usually based on a limited portion of news coverage, within the traditional frame of content analysis. TIPS aims at extending this perspective, by making reference to the whole set of newspaper articles dealing with relevant technoscientific issues.

There are some methodological problems that need to be addressed:

- The longitudinal character of the observed phenomena.
- The importance of a comparative perspective both in diachronic and in synchronic terms.
- The “demarcation problem”, that is what distinctive traits make science a specific object being studied; that allows the thematic domain of technoscience to be defined.

In order to address the first two problems, we designed a methodology and a system to collect, manage and provide access to newspaper articles published in diverse languages and over a long time span. With regard to the third problem, we opted for a “pragmatic” approach assuming the point of view of a hypothetical “typical newspaper reader”, asking ourselves what this person may recognise as “science”. That led us to a list of six features which should characterize a technoscience-related article:

- a scientist/engineer is mentioned;
- a research centre is mentioned;
- a scientific journal is mentioned;
- a scientific discipline is mentioned (excluding the humanities and social sciences);
- there is a generic reference to research processes and/or technological innovations;
- a discovery, an innovation, a scientific instrument or a medical apparatus is mentioned.

These features will be adopted to build a manually labeled corpus used to evaluate some of the methodology steps and gain insights on some research questions.

## Methodology

The first step in the design of the methodology and the system was the analysis and the abstraction of (some) research methodologies adopted by the sociologists to investigate their research hypotheses. “Objects” used to carry out these investigations are generic informative resources (e.g. a newspaper article), hereafter named *document*s. Documents are organized in *source*s. With regard to on-line newspapers, a source can be a section of a newspaper, e.g. “Homepage”, “Technology” or “Science” section. Sources can be organized in *source set*s: for instance, a newspaper is the source set constituted by all the sections in which the articles are published, or by a meaningful subset of these. Sources could be organized in a hierarchy of source sets; currently, source sets are grouped according to different national and cultural contexts, e.g. “English newspapers”.

The analysis of the research methodologies allowed us to identify some common steps:

1. consider a theme, e.g. “science and technology” or “science”;
2. identify (classify) documents pertinent to the theme;
3. consider a specific issue; an issue could be a sub-theme (a more specific theme within a theme, e.g.“nuclear power” in the “science and technology” theme) or can be another theme (e.g. “political debate on infrastructure”).
4. identify documents pertinent to the theme and relevant to the considered issue; those documents constitute the sub-corpus of interest to investigate the research hypothesis;
5. perform analyses on the identified sub-corpus, e.g. how the presence of a theme in the corpus varies over time

The sub-corpus of interest could be also the entire document corpus – e.g. the entire set of articles gathered from the Italian newspapers; in that case, no specific issue is considered and steps 3–4 are not performed.

The analyses on the sub-corpus usually include:

- exploration of the sub-corpus, that could be completely manually or supported through unsupervised or supervised techniques;
- extraction of sub-themes and study of their evolution through time;
- study of the variation of the distribution of words, named entities or linguistic properties in the sub-corpus, eventually by the comparison with the overall corpus;
- computation of indicators trend.

Indicators are meant to provide a measure of the degree to which a certain concept/aspect is present in the considered (sub)corpus. Looking at the trends of the indicators the researcher can easily identify source sets, sources or periods with peculiar variations, e.g. peaks, and inspect the subsets of the corpus related to these variations in order to gain a deeper comprehension of their causes. The research methodologies are not necessarily linear – from step 1 to step 5 – but they can involve an iterative process where we can reconsider, for instance, the research question or the identification of the (sub)corpora of interest. The analysis of the indicators trends could be the step that leads to a new iteration.

The remainder of this section will describe the diverse methodology steps.

### Source Set Selection

Setting up the source set required some crucial methodological choices: how to collect the articles and the choice of the newspapers.

Regarding the former, our aim was to start up a long-lasting monitoring platform which could provide a real-time automated monitoring system rather than a static document collection. In order to achieve durability and automatic real-time updating, we opted for the on-line version of newspapers, collecting the news-feeds through RSS.

As for the choice of the newspapers, we decided to follow two main criteria, defined as the *popularity* and the *spin* of the newspapers. Popularity has been operatively translated into the average number of daily readers, while the spin concerns the cultural and political orientation of the newspaper. When considering Italian newspapers, we relied on the Audipress 2010 data on popularity. We excluded the most popular newspaper, being almost exclusively a sport newspaper. We considered those ranked immediately below: “Il Corriere della Sera”, “La Repubblica”, “La Stampa” and “Il Sole 24 Ore”. Then we added four additional newspapers considering two additional criteria: the cultural and political orientation and the geo-political location.

### Document Collection

Once the newspapers of the source set are chosen, their RSS feeds are identified with manual inspection to their websites combined with an automated XML feeds survey through website crawling. This approach requires to periodically check the source collection coherence, in case some new RSS feed is published or some other is cancelled. It is known that paper and on-line editions might contain different sets of news; moreover, not all the news published on-line may be advertised. We have to accept these limitations, bearing in mind that following RSS feeds rather than performing a full website crawling responds to the (near) “real-time” collection requirement inherent in our main research purposes. The entire set of selected RSS feeds constitute our collection of sources. In case RSS feeds are absent or discontinued we shift to HTML website traversal, selecting newspaper main sections as a base for crawling.

The newspaper document harvesting process consists of three main phases: proper article collection, scraping, and de-duplication.

In the first phase, when the articles are downloaded, all RSS feeds in our source collection are continuously scanned several times a day, and feed items are collected along with their metadata. A single item consists of: newspaper name and feed, collection date, feed publishing date, feed content (usually a summary of the full newspaper article, or its first lines), URL of the full article and, sometimes, name of the author. Subsequently, the HTML page referenced by the feed URL is retrieved, along with its metadata.

The scraping process consists in extracting the relevant part of the article text from the HTML source, discarding ads, scripts, banners and any other content that is not within the boundaries of the article itself and out of our research scope. We explored several strategies; the currently adopted approach mainly relies on the Newspaper3k open source library.^1^ As a complement and fallback, html2text scraping library is used.^2^

It may happen that an article is published in several different feeds. While its presence in multiple feeds adds significance to that article and represents a valuable information, we have to ensure that the article appears only once in the corpus. For this reason we scan for duplicate articles and retain only one version, keeping track of the different news-feeds involved. It may also happen that different newspapers share the same article. Another frequent case of duplicate item collection happens with articles being updated over time. In this case we keep the last version of the article, saving a record of the publication dates and times of each version. A refined version based on near-duplicates detection is currently being tested.

The source code of our collection, scraping and deduplicating platform is published with AGPL open source license.^3^

### Document Classification

After de-duplication, articles are classified according to their pertinence to themes and sub-themes. For each theme, we investigated text classifiers that can be considered instances of Knowledge Engineering (KE) classifiers [19], where a set of rules encoding expert knowledge are manually defined.

Our classifiers rely on a list of weighted descriptors and a threshold $\theta$; each descriptor can be either a single word or a set of words. A score is assigned to each document on the basis of the weight of those descriptors occurring in the document; if the score is equal or greater than $\theta$, the document is labelled as pertinent to the theme; otherwise, it is marked as not pertinent. Each classifier is characterized by two groups of descriptors: *keyword*s and *multiplier*s. Keywords are specific descriptors, i.e. words or sets of words that are determinant in distinguishing whether a document deals with a certain theme or not. In the specific case of the thematic area of technoscience, keywords have been manually identified according to the following criteria:

- Names of research institutions and scientific journals which are frequently cited by newspapers;
- Names of scientific disciplines;
- Words frequently associated to scientific research activities (such as those regarding discoveries, scientific achievements, features of the laboratory work and scientific instruments);
- Words frequently associated to technological innovations, including those which can be considered as consumer products, like smart-phones, PCs, the Internet, appliances, means of transport.

Differently from keywords, multipliers are descriptors related to the thematic area, but less specific than keywords. Some examples of multipliers we have considered for the field of “technoscience” are “research”, “discovery’, “environment”. The multipliers only count when an article contain at least one keyword. More formally, if *K* and *M* denotes respectively the list of keywords and multipliers, and if $w_k$ is the weight associated to keyword $k \in K$ and $w_m$ is the weight associated to the multiplier $m \in M$, the score $s_d$ of a document *d* is computed as:where $t_{d,k} = 1$ if the keyword *k* appears in the document *d*, $t_{d,k} = 0$ otherwise; similarly $t_{d,m} = 1$ if the multiplier *m* appears in the document *d*, $t_{d,m} = 0$ otherwise. The current technoscience classifier uses 218 keywords (with weights ranging from 2 to 15) and 17 multipliers (all weighting 2). Keywords and multipliers, as well as their weights, have been selected and applied after manually comparing the performances of many different versions of the classifier, by adding or dropping words, changing their weights and shifting words from the keyword to the multiplier group. The threshold has been identified by looking at the distribution of the scores obtained from the textual analysis, and choosing the value which could best distinguish between pertinent and non-pertinent articles; the currently adopted value is $\theta =20$.

When no multipliers occur in the document, the classification function is a linear threshold function with threshold $\theta$; when at least a multiplier occurs in the documents, the classification function corresponds to a *polynomial discriminator of order 2* [1]. Indeed, the above function can be described by a multinomial equation of degree 2 in the variables $x_{1}, \dots , x_{|K|}, x_{|K|+1}, \dots , x_{|K+M|}$ where

- $x_{1}, \dots , x_{|K|}$ are the keywords, $t_{d,k}$’s
- $x_{|K|+1}, \dots , x_{|K+M|}$ are the multipliers, $t_{d,m}$’s
- the weight associated to $x_i^2$ is always zero
- the weight associated to $x_i x_j$ is always zero if both *i* and *j* are in $\{1, \dots , |K|\}$ (i.e. they correspond to keywords) or are in $\{|K|+1, \dots , |K+M|\}$ (i.e. they correspond to multipliers).

In order to investigate the effectiveness of the proposed classifier, we manually labelled a sample of documents disjoint from that used to extract keywords and multipliers and to set the threshold $\theta$. We extract all the articles published in the four most popular newspapers mentioned in Sect. 3.1 after 2008-01-01 and before or on 2014-09-04, satisfying the query “scienz*”.^4^ That led to 7779 possibly relevant articles; a random sample was extracted from this subset and manually labelled according to the features/criteria described in Sect. 2; documents were considered relevant if at least two criteria were met. That results in 1167 documents related to technoscience. Then, two random sets of articles with a score ranging from 1 to 19 – i.e. articles classified as non-related to the technoscientific theme, hereafter denoted as “non-relevant documents” – were selected from the corpus. The size of the first random sample was computed in order to maintain approximately the same size of the random sample of documents above the threshold. The second sample of non-relevant documents was obtained by augmenting the fixed non relevant sample by additional randomly selected documents with a score below the threshold of relevance; the number of added documents was computed in order to obtain a final sample where the proportion of documents above and below the threshold was approximately the same as in the entire corpus. Both non-relevant samples were labelled on the basis of the 6 criteria described in Sect. 2. In this way we obtained two datasets: one with 3814 documents (relevant sample + fixed non-relevant sample) and one with 5121 documents (relevant sample + proportional non-relevant sample).

As for the evaluation, we considered the KE classifier and two supervised learning approaches:

- **SVM:** Linear Support-Vector Machine (SVM) using all the terms occurring in the vocabulary of the training sets; each term was represented by TF$\cdot$IDF, where TF is the *Term Frequency* and IDF the *Inverse Document Frequency*.
- **NB:** Naïve Bayes (NB) classifier using all the terms occurring in the vocabulary of the training sets; each term was represented by the TF.

We investigated also SVM and NB with document representation based only keywords and multipliers: **SVM-Vocab** and **NB-Vocab**. The former used IDF as term feature, while the latter the (binary) occurrence of the term in the document; the motivation was to use the same information of the KE classifier.

The SVM and the NB classifier implementation is that made available in Apache Spark (version 2.2.0). The evaluation was performed using a 60/40 split for the training/test set. As for the KE classifier, we used that trained by trial and error by the sociologists.

Table 1 reports the results in terms of F1 on the test set for the most effective combinations of methods and representations.

**Table 1.** F1 for the diverse classifiers and splits.

Method	Feature	F1			
		Random Split		Time Split	
		Fixed	Prop	Fixed	Prop
SVM	TFIDF	0.9153	0.9208	0.9098	0.8892
SVM-Vocab	IDF	0.9268	0.9297	0.9230	0.9226
NB	TF	0.9300	0.9227	0.9175	0.9074
NB-Vocab	Bin	0.9168	0.9020	0.8983	0.8941
KE	–	0.9286	0.9428	0.9298	0.9397

Automatic text classifiers can achieve an accuracy comparable to that of the KE classifier, whose development required a great effort by the sociologists; for this reason, we are currently relying on supervised learning approaches, e.g. SVM and Random Forests, to build additional classifiers, e.g. those for newspapers in other languages. The main issue with the previous experimental methodology is that articles in the corpus have a chronological order and, in principle, we should train the classifier on the past and predict categories in “future” documents. Following the approach reported in [12], we split the dataset chronologically; a 60/40 training/test split was adopted. The results are reported in the last two columns of Table 1: the KE classifier is still the most effective and it is less affected by the chronological split, thus suggesting that is capable to generalize through time. The curated vocabulary created for the KE classifier seems to be the reason for the robustness: indeed, also SVM-Vocab is only slightly affected by the use of a chronological split.

### Indicators

Indicators are meant to provide a measure of the degree to which certain properties characterize a set of documents related to a theme or specific issue. The set of documents can be the entire corpus or a subset identified for the investigation of a specific research hypothesis. We defined several indicators; three of them are reported below:

- *salience*: ratio between the number of articles classified as relevant for a specific theme and the total of articles published over the same period by the source;
- *general framing*: distribution of the relevant articles across the various sections of the news websites;
- *risk*: presence in an article text of a set of words associated with the risk domain.

The first two indicators have been introduced in order to have two different measures of the coverage of technoscientific contents in the news. These indicators are normalized over the number of published articles – in the period or in the source – in order to obtain relative measures not affected by increments of articles published in absolute terms.

The last indicator, *risk*, is rooted in the “risk society” notion originally developed in the sociological field. The risk society refers to the idea that within contemporary society technoscientific issues are imbued with fears and preoccupations about unforeseen effects, calling for a precautionary approach on the side of policy making, society, and the public [2]. Diverse instantiations on the risk indicator have been proposed in [17] and [4]. The currently adopted version relies on term weight computed for keywords in a controlled vocabulary.

### Information Access and Retrieval

All the collected newspaper articles are enriched with the scores assigned by the classifiers and some of the indicators, e.g. the risk indicator. All this information along with the extracted content and metadata, is stored using a NoSQL Database Management System, more specifically MongoDB; this technology has been selected because of its capability to scale and to handle replicas. All the information in the databases is indexed and made available through a Search Server implemented with Elasticsearch.^5^ This search server relies on the Open Source library Apache Lucene and allows the user information need to be expressed through fulltext-search queries and, if needed, through the Lucene Query syntax which includes the specification of boolean constraints, fuzzy queries (e.g. “scienz*”) or proximity queries. This component is crucial in our application domain where the users are researchers, mostly sociologists experts in the study of how technoscientific issues are discussed and represented in the mass media. The information retrieval functionalities are used:

- to get articles most pertinent (top-*k*) to themes, e.g. technoscience, on the basis of the classifier score and other metadata, e.g. a time range or the fact that the articles were published in Homepage;
- to identify subsets of the corpus pertinent to specific technoscientific issues, e.g. using *standing queries* [13];
- to perform fine-grained analysis of specific issues, looking at different perspectives/angles on the basis of complex queries devised by expert users.

Documents can be ranked according to several criteria, e.g. recency, classifier score, date range, newspaper, newspaper sections were they appeared, topicality “estimated” by BM25 [18].

## The TIPS Architecture

**Fig. 1.**
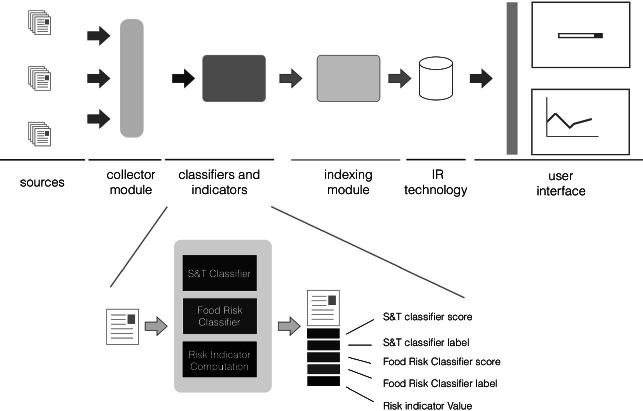
TIPS Architecture.

The methodology has been implemented in an information system called TIPS^6^; a very concise description can be found in [4, 8]. Since TIPS has been designed and developed for research purposes, it responds to several design criteria: modularity, open source components and languages, reproducibility of results, measurability of process, data and system reliability, data safety. A pictorial representation of the main modules constituting TIPS is reported in Fig. 1. The sources are RSS feeds of newspapers sections; however, we have recently extended the types of sources, e.g. including blogs and tweet streams. The collector module implements the procedures described in Sect. 3.2. The classifiers and indicators module performs the classification using the techniques described in Sect. 3.3 and computes some of the indicator values, most specifically those that can be computed for each document like the risk indicator [4]. The indexing module and the IR technology refer to the IAR functionalities discussed in Sect. 3.5. The last modules are those responsible to expose all the services as Web API – that can be access only through authentication – and the Web User Interface (Web UI).

TIPS has been designed to support reproducible research. Each researcher can work on a number of projects. A number of classifiers and predefined issues is associated to each project; a researcher can define its own issues, which are translated into structured queries that can consider both constraints on the full-content of the documents and/or their metadata. The researcher can then inspect trends of the indicators computed on the defined sub-corpus, and search the corpus in order to access the documents and carry out a more fine grained analysis. Several interactions with the system (task definition, search activities) are logged.

**Fig. 2.**
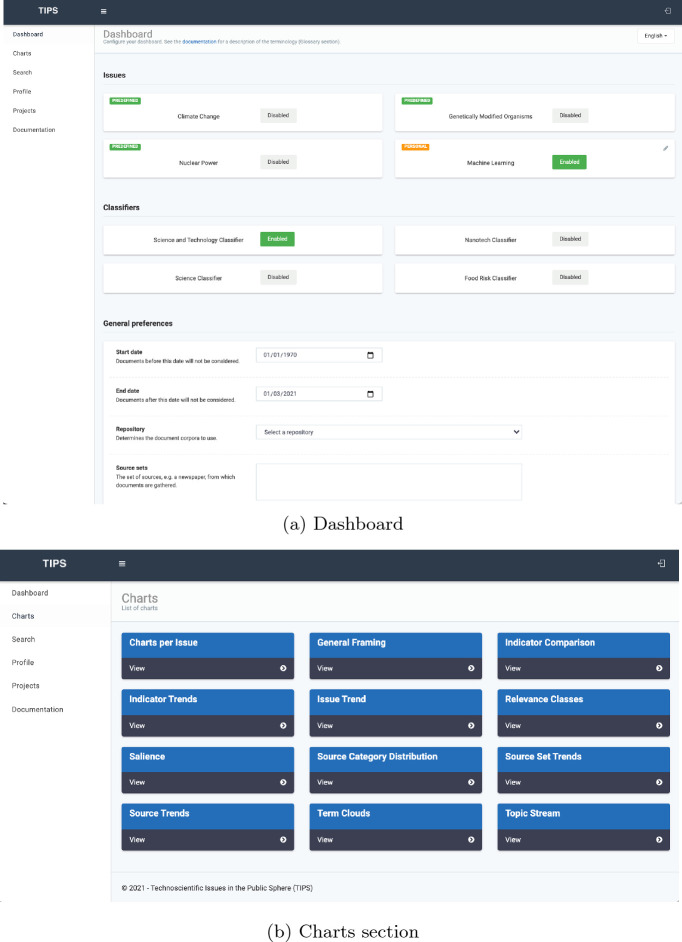
TIPS Web UI

As mentioned above, TIPS functionalities are accessible through a Web UI. The UI has a dashboard section which is the page when a user is redirected after the login – see Fig. 2a. The dashboard allows the researchers to specify the classifiers and the specific issues to be used for their investigation. The specification of an issue determines the sub-corpus of interest (e.g. only documents topically relevant to “climate change”). The researcher must specify the document repository for the analysis. Currently, there are several repositories, e.g. that including all the Italian Newspapers, that including all the English Newspapers, diverse longitudinal samples or newspaper corpora spanning several decades and gathered through available archives.

Once the document repositories, classifiers and issues have been specified, the researcher can access charts that show indicators trends and/or indicator comparisons; charts are dynamically computed on the basis of the selected repository, classifiers and indicators. The currently available charts are displayed in Fig. 2b which is a screenshot of the “Charts” section of the TIPS Web UI; the first chart allows access to charts generated using the sub-corpus of documents relevant to a user-specified issue. In the following section we will discuss how the TIPS Architecture allowed us to answer several research questions through the analysis of some indicators.

## Following Technoscience in the News

A first result that we obtained concerned with the trend of documents relevant to technoscientific issues. We considered a subset of the newspapers and look at the number of relevant documents over the years; the trend seems to suggests that the tendency on publishing articles on technoscience has become stronger over the years. However, because we gathered all the published articles, we were able to compute the *salience indicator* that showed how the coverage of technoscience is more stable and the increment is mostly due to the increasing number of articles published on-line over the years. This result is documented in [14].

**Fig. 3.**
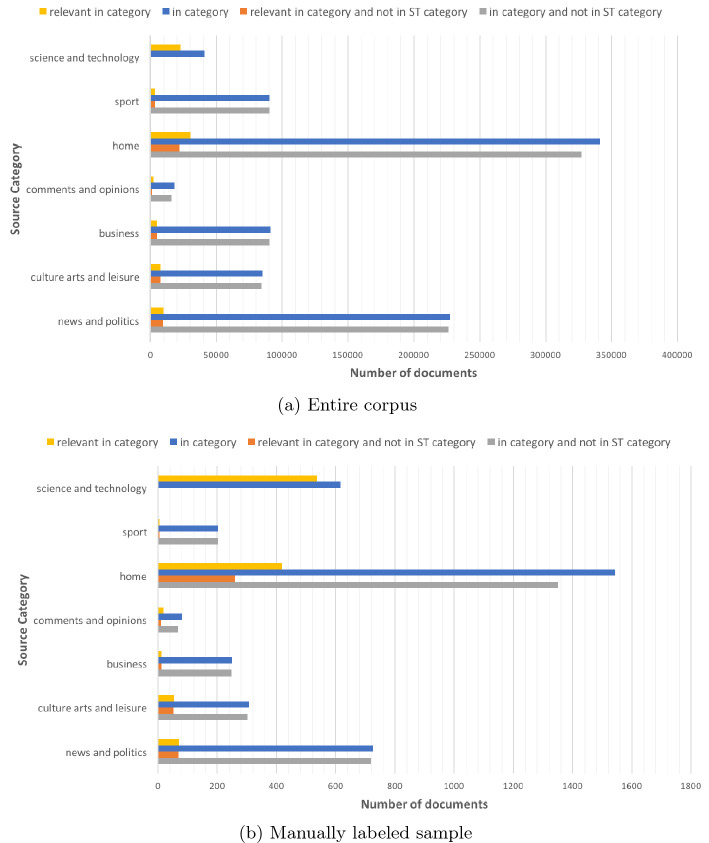
Relevant articles per source category in the entire document corpus (Fig. 3a) and in the manually labeled sample (Fig. 3b).

Automatic classification of technoscientific content was investigated as an alternative to the classification performed by the newspaper editors, e.g. that derived by manually assigned tags, if any, or the section(s) where the articles were published. We could have classified as relevant all and only the articles published in “Science” or “Technology” sections of the newspapers. However, we are not aware of the criteria adopted by newspaper editors and we conjectured that content relevant to technoscience following our criteria is spread over all the section categories. In order to check if using newspaper-provided classification could have prevented us from identifying some relevant articles, we computed the *general framing* indicator. Seven different source categories were manually defined by grouping newspaper feeds according to their content: “science and technology”, “sport”, “home” (homepage), “comments and opinions”, “business”, “culture, art and leisure”, and “news and politics”. Figure 3a reports the number of articles per source category, the number of those articles classified as relevant to technoscience; the indicator shows how technoscientific content is published also in sections not in the “science and technology” category. Since some articles could have been published both in a “science and technology” section and in other sections, we reported also the number of articles published in a given category but not published in a “science and technology” category; also in this case, the number of relevant articles in other source categories is positive. We performed the same analysis on the manually labeled sample described in Sect. 3.3—the “fixed” sample was used. The result of the analysis is reported in Fig. 3b and shows that also in this case there is a positive number of relevant documents published in feeds not in the ‘science and technology” category. These results support our intuition of not relying on the newspaper classification.

*Salience* and *general framing* provide us with quantitative measures of the coverage of technoscience in the newspapers, and the distribution of technoscientific content in different sections (categories). The latter could provide an indication on the thematic frames within which technoscientific issues are discussed—e.g. business, culture, politics. In order to gain additional insights on the thematic frames, we could follow one of the ways sociologists ordinarily use to analyse texts: “read texts and produce interpretations based on insights their readings [$\dots$] produce a set of themes, create a coding sheet, and then code texts by reading them [$\dots$] search texts for keywords and comparing subsets of texts with respect to the prevalence of those keywords” [5]. However, as suggested in that work, these approaches are impracticable for large number of documents or requires to restrict the scope of exploration a priori.

For this reason, we exploited the Topic Modelling algorithm Latent Dirichlet Allocation (LDA) [3]. We used the approached proposed in [22] and implemented in Mallet,^7^ we extracted 20 topics from the articles classified as relevant in the entire dataset and we manually labelled them according to the top 100 words extracted for each topic.^8^ Table 2 reports 3 of the 20 extracted topics; sixteen of these topics with the top 100 keywords have been made available online.^9^ The first column of the table reports the manually assigned label, while the second column reports the topic proportion per year; topic proportion was computed counting the number of words in each topic published in a given year and then normalizing over the total number of words for that year.

The analysis of the trends of the topic proportions showed that the coverage of some thematic issues tend to be constant over the years. This is the case of research concerning with astronomy or space exploration. Peaks are present respectively in 2012 and 2014. The former can be explained by the involvement of the European Space Agency in multiple missions, e.g. the launch of Vega, or the discovery made through the Fermi Gamma-ray Space Telescope on the Extragalactic Background Light. In 2014 there were multiple achievements in space exploration, e.g. the missions by India and Japan, the Lunar mission by China, the first landing on a comet (67P/Churyumov-Gerasimenko), or the observations provided by the NASA’s Mars Curiosity rover; moreover, 2014 was the year of the first Italian woman in space. Other topics present more prominent peaks. This is, for instance, the case of topic labelled as “research policies” where a peak in term of topic proportion is present in the 2010. The peak can be explained by two controversial cases also evident from the top terms: the *stamina therapy* and the *university reform*. The first case refers to a controversial stem-cell therapy that had media and political repercussion; the second case refers to the reform concerning the reorganisation of universities in Italy.

**Table 2.**
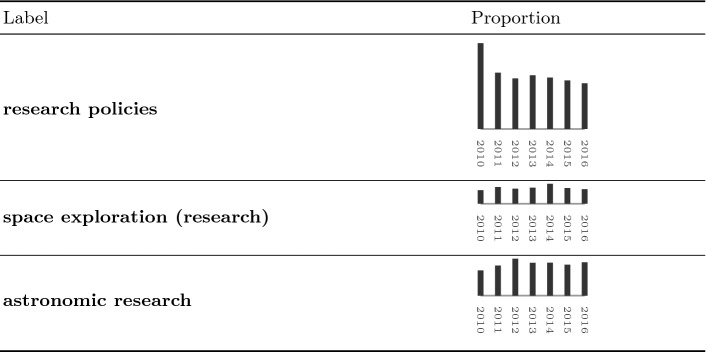
Three of the twenty topics extracted from technoscientific documents. The three charts use the same scale for the *y* axis.

## Related Works

Previous works on news search engines and media monitors are relevant to the work reported in this paper. In [9] the author described the architecture of a news search engine, *Velthune*; our system shares with Velthune the adoption of RSS and classifiers to complement the classification provided by the newspapers, e.g. the distribution among the newspaper sections. Differently from Velthune, our system was specifically designed to support expert users such as sociologists in the investigation of their research hypotheses. The Web UI has been designed in order to support research methodologies via diverse forms of interaction which include the creation of subcorpora on the basis of full-text and metadata, and the visualisation and the interaction with indicators and extracted topics. Even if also Velthune allows a private set of queries to be recorded, in TIPS the recorded queries are the basis for the analysis performed by the experts; all the indicators and visualisation are dynamically generated on the basis of the subcorpus determined by the query (issue). Our starting point was the methodological premises of our expert users, their methodology and how to support them in their exploration.

The Europe Media Monitor (EMM) [20] is a suite applications developed to monitor the media in order to update its users on relevant developments; EMM users include EU institutions, national organizations in the EU Member States and in some non-EU countries, international organizations, as well as the general public. The NewsBrief application is the most relevant to the work reported in this paper. One of the peculiar features of EMM is that supports Multilingual Information Access and Retrieval (IAR).

NOAM [6] and the system described in [7] are also relevant to our work. They were designed and developed in order to automate media content analysis for the social sciences. They rely on a modular architecture, allow diverse type of sources to be monitored, and are equipped with mood and topic detector, sentiment extractor and readability and popularity annotators.

TIPS shares with EMM part of the architecture components; with NOAM the focus on Social Science. However, TIPS is focused on the research areas of PCST and STS, is equipped also with classifiers that have been developed to fulfil specific theoretical principles underlying the study of S&T and provides diverse visualizations of the news corpora based on novel indicators. TIPS allows the information need to be described through a rich query language: this functionality is particularly useful to support expert users such as sociologists in the investigation of their research hypotheses. Moreover, TIPS supports also analysis and exploration of the information space by exploiting the thematic structure, e.g. through the adoption of topic modelling algorithms.

## Final Remarks

In this paper we introduced a methodology that was co-shaped by sociologists and computer scientists to support the investigation on public perception of technoscience. We relied on a pragmatic approach based on six criteria to deal with the problem of demarcation. We showed how the existing newspaper categorizations are not sufficient to capture all the technoscientific content and how automatic classifiers can be adopted to obtain satisfactory results, even in evolving corpora. We defined several indicators and discuss how they can help us to avoid distorted interpretations of the prominence of technoscientific content in the news, e.g. through the salience indicator.

The adoption of machine learning algorithms to unveil the thematic structure, e.g. LDA, allowed us to rapidly obtain a general view of the public discourse on technoscience and its evolution; that would have been impossible (in reasonable time) through manual inspection.

The methodology and the TIPS system were the basis for carrying out other research activities, e.g. on the energy transition case in Italian daily newspapers [16] and to track biomedicalization in the media [15]; in the latter work, a comparative study between Italy and UK was performed and the proposed methodology relies both on topic analysis through time and the risk indicator.

Our current research directions include the study of heterogeneous sources, e.g. tweet streams, blogs, and forums, along with methods to study additional dimensions to characterize the public discourse on technoscientific issues in the media. Those methods include approaches to “measure” document readability and to characterize, e.g. through patterns of syntactic features, the language used for the communication of science and technology.

Moreover, we are exploring languages to support researchers, experts in domains other than computer science, in carrying out their research tasks, e.g. following the idea suggested in [23], where Zhai proposed to go beyond search and toward a general analysis engine that can support, for instance, decision making, learning or other tasks.

## Footnotes

1 https://newspaper.readthedocs.io/.

2 https://github.com/Alir3z4/html2text/.

3 https://gitlab.com/mmzz/hactar.

4 The word “scienza” is the Italian translation for “science”; the notation “scienz*” refers to all the words that begins with the string “scienz”.

5 https://www.elastic.co/elasticsearch/.

6 https://www.tipsproject.eu/tips.

7 https://github.com/mimno/Mallet/releases/tag/v2.0.8RC3.

8 We used 17 as random seed, 5000 iterations and 10 as optimization interval.

9 http://www.dei.unipd.it/ dibuccio/tips/topics.html.
